# How are HIV services in the UK and Ireland managing care home residents living with HIV?

**DOI:** 10.1007/s41999-025-01389-4

**Published:** 2025-12-19

**Authors:** Benjamin Moshy, Tristan J. Barber, Maithili Varadarajan, Marta Boffito, Clea Tanner, Jaime H. Vera, Gillian Farrell, Emma Devitt, Alison Grant, Maria Liu, Charlene Goffe, Borja Mora-Peris, Laura Hilton, Howell T. Jones

**Affiliations:** 1 Ian Charleson Day Centre, Royal Free Hospital, Royal Free London NHS Foundation Trust, Pond Street, Hampstead, London, NW3 2QG UK; 2 Institute for Global Health, University College London, London, WC1E 6JB UK; 3 Kobler Clinic, St Stephen’s Centre, Chelsea and Westminster Hospital, 369 Fulham Road, London, SW10 9NH UK; 4 Department of Infectious Disease, Imperial College London, South Kensington Campus, London, SW7 2AZ UK; 5 Department of Global Health and Infection, Brighton and Sussex Medical School, University of Sussex, East Sussex, Brighton, BN1 9PX UK; 6 The Lawson Unit, Royal Sussex County Hospital, Brighton and Sussex University Hospitals, Eastern Road Brighton, Brighton, BN2 5BE UK; 7 The GUIDE Clinic, Department of Genitourinary Medicine and Infectious Diseases, St James’s Hospital, Dublin, D08 NHY1 Ireland; 8 Harrison Wing,, Guy’s and St. Thomas’ NHS Foundation Trust, Great Maze Pond Road, 2nd Floor, Southwark Wing, London, SE1 9RT UK; 9 Jefferiss Wing Centre For Sexual Health, St Mary’s Hospital, Praed Street, London, W2 1NY UK; 10 The Number 22 Clinic, Mid and South Essex NHS Foundation Trust, Prittlewell Chase, Westcliff-On-Sea, Essex, SS0 0RY UK

**Keywords:** Ageing, Care home, Frailty, HIV, Long-term care, Polypharmacy

## Abstract

**Purpose:**

The average age of people living with HIV in Europe is rising and with it the likelihood of entering long-term care. Little is known about the characteristics of people living with HIV in care homes and how their HIV care is delivered.

**Methods:**

We conducted a multi-site, cross-sectional descriptive evaluation of care home residents receiving HIV care at seven centres in the UK and Ireland. Data included demographics, HIV history, comorbidities, medication use and HIV service contacts over the preceding 12 months.

**Results:**

The sample included 62 residents with a median age of 61 years (33–92); 81% were male, 56% heterosexual and 60% born in the UK or Ireland. Most (92%) were virally suppressed, having lived with HIV for a median of 21 years and in care homes for 3 years. Multimorbidity, frailty and polypharmacy were common. Care home residents represented 0.2% of total service users across all sites. Over 12 months, participants had a median of four HIV service contacts with two HIV viral load tests. Models of care varied substantially between centres, highlighting inconsistent approaches to supporting this population.

**Conclusion:**

This service evaluation describes a small, heterogeneous cohort of people living with HIV in long-term care across the UK and Ireland. Participants commonly had complex health needs, and HIV services used varied approaches to deliver care. These descriptive findings provide a foundation for larger studies to examine care models and outcomes for this group to inform future guidance.

**Supplementary Information:**

The online version contains supplementary material available at 10.1007/s41999-025-01389-4.

## Key summary points

**Aim:**

To describe the demographics and clinical characteristics of care home residents with HIV accessing care at seven centres across the UK and Ireland and to explore how their HIV care is currently delivered.

**Findings:**

People living with HIV who reside in care homes are a diverse group with varied needs; within this cohort, multimorbidity, frailty and polypharmacy were common with HIV care models varying across services.

**Message:**

Although care home residents currently represent a small proportion of people living with HIV at participating centres, their number is likely to increase in the coming years. We must develop strategies at a Eurpean level that meet the complex ageing and HIV care needs of this population.

**Supplementary Information:**

The online version contains supplementary material available at 10.1007/s41999-025-01389-4.

## Introduction

In 2021, roughly half of all people who accessed HIV services in England were aged ≥ 50 years, and this proportion is projected to rise to around 70% across Europe by 2030 [1, 2]. Meanwhile, one in six new HIV diagnoses in Europe occurs in a person aged over 50 [3]. People living with HIV experience high rates of multimorbidity, frailty and polypharmacy and therefore must increasingly manage health and social issues related to ageing alongside HIV, often across fragmented primary, secondary and tertiary care services [4, 5]. People living with HIV also experience stigma and discrimination at higher rates than the general population [6]. Person-centred, integrated care is associated with better health outcomes and may reduce the need for long-term care [4, 7].

The Organisation for Economic Co-operation and Development (OECD) defines long-term care as integrated care provided to people who need continued assistance due to chronic activity limitations or functional dependency, delivered in care homes, day hospitals or home settings [8]. In the UK and Ireland, long-term care facilities include residential homes, which operate without a qualified nurse on site, and nursing homes, where nurses are present [9]. Since 2011, the proportion of care home residents living in nursing homes compared to residential homes in England and Wales has increased, reflecting rising levels of dependency and frailty [10]. Despite extensive data on the general care home population, little is known about care home residents living with HIV in Europe.

Guidelines produced by the European AIDS Clinical Society (EACS) highlight the importance of identifying and managing frailty in people living with HIV [11]. In some European countries, geriatricians are involved in specialist services for older people living with HIV, but such models usually rely on attendance at outpatient clinics, which may not be feasible for those living in care homes [12, 13].

The Care for HIV and Ageing Implementation Network, UK and Ireland (CHAIN-UKI), is a collaboration of clinicians focused on improving care for people ageing with HIV across the UK and Ireland. Representatives from seven HIV services participated in this work; each site coordinates HIV care for at least one person residing in long-term care.

This multi-site service evaluation aimed to describe the demographics and clinical characteristics of people living with HIV who reside in long-term care facilities in the UK and Ireland. The study also sought to document how HIV care is currently delivered to care home residents, including patterns of monitoring, consultation models and multidisciplinary involvement.

## Materials and methods

### Evaluation design

We undertook a cross-sectional descriptive evaluation across seven HIV services in the UK and Ireland. In the UK, HIV services are required to supply data to the HIV and AIDS Reporting System (HARS), maintained by the UK Health Security Agency [14]. HARS is used to identify trends and inform public health policy with all UK HIV services maintaining local databases of required data [14]. At the six UK sites, clinicians searched their local databases along with electronic patient records to identify adults living with HIV who were resident in long-term care. In Ireland, where there is no equivalent central HIV registry, the participating site identified eligible care home residents by review of local electronic patient records. Data was then extracted and anonymised before analysis. Finally, the site representative at each service completed a brief structured survey describing their current model of HIV care for residents in long-term care.

### Evaluation population

Inclusion criteria were: adults (≥ 18 years) living with HIV-1 or HIV-2, registered for care at a participating HIV service and residing in long-term care (residential or nursing homes) in August 2024. Residents of sheltered housing, extra-care sheltered housing or their own home with visiting carers were excluded. Each site also reported the total number people accessing care at their service to determine the proportion that were care home residents.

### Evaluation assessments

A range of variables were extracted including personal demographics such as age, gender, ethnicity and country of birth, as well as HIV-related information including numbers of years living with HIV, most recent viral load, current and nadir CD4 counts, CD4:CD8 ratio and any history of AIDS-defining conditions.

Data was also collected on the length of time each person had lived in a care home, their comorbidities and overall multimorbidity profile, and their medication use, encompassing both antiretroviral therapy (ART) and other prescribed medication. Cognitive status was recorded through documented diagnoses of dementia in either primary or secondary care records. Multimorbidity was summarised using the Charlson Comorbidity Index (CCI) [15]. Frailty was classified using the Clinical Frailty Scale (CFS) [16, 17]. The CFS is not part of routine HARS data collections. CFS scores were derived retrospectively from clinical documentation, including functional, cognitive and comorbidity information. Polypharmacy was defined as taking five or more regular non-ART medications [18]. Anticholinergic burden was estimated using the online Anticholinergic Cognitive Burden (ACB) calculator, which combines scores from the anticholinergic cognitive burden and German anticholinergic burden scales [19–21]. An ACB score ≥ 3 was considered high.

Finally, information on HIV service usage over the preceding 12 months was reviewed and included any scheduled or unscheduled clinical contact related to HIV care, including: in-person outpatient reviews, telephone or video consultations, or community visits to care homes.

For the service-level survey, clinicians at each site completed a ten point questionnaire detailing current practice for people living with HIV in care homes, including modes of consultation (in-person, virtual, community visits), involvement of different professionals and participation in multidisciplinary meetings. The data collection tool is available in Appendix 1.

### Ethics

As this was a service evaluation, formal research ethics committee approval was not required, in line with UK Health Research Authority guidance. The project was registered at each participating centre with the respective clinical governance team. All collected data was anonymised in line with Caldicott Principles before leaving each site. Reporting of demographic data and outcomes was aggregated to minimise the risk of identifiability.

### Statistical analysis

Data was summarised using descriptive statistics. Categorical variables are presented as frequencies and percentages; continuous variables as medians with ranges or interquartile ranges, as appropriate. Given the small sample size and heterogeneity of the cohort, no inferential statistical comparisons between models of care were undertaken.

## Results

A total of 62 participants across the seven sites met the inclusion criteria. The number of care home residents per site ranged from 2 to 14 (Table 1). Across all sites care home residents represented 0.2% of total service users whereas they represented < 1% at each individual site. Data was grouped into five domains (Table 2). The cohort was demographically diverse, with a wide age range and a predominance of men. Most particpiants had long-standing HIV with high levels of multimorbidity, frailty, polypharmacy and substantial anticholinergic burden. Models of HIV care for care home residents were heterogeneous and in the absence of specific guidance had largely evolved locally.

**Table 1:** Number of care home residents per evaluation site

HIV clinic	Total number of service users at each site	Number of care home residents accessing HIV care at each site	Percentage of care home residents in each service (%)	Percentage of care home participants from each site in total sample (%)
Chelsea and Westminster NHS Foundation Trust, London, UK	12,500	14	0.11	23
The Lawson Unit, Brighton, UK	2500	13	0.52	21
The GUIDE Clinic, Dublin, Ireland	3300	13	0.39	21
Ian Charleson Day Centre, London, UK	3100	8	0.26	13
Harrison Wing, London, UK	4800	7	0.15	11
Jefferiss Wing, London, UK	3500	5	0.14	8
The Number 22 Clinic, Essex, UK	1500	2	0.13	3
Total	31,200	62	0.2	100

**Table 2:** Summary of demographic and clinical characteristics

Clinical characteristics (*n* = 62)	Results
Demographics	
Age: years (median; range)	61 (33–92)
Male, *n* (%)	50 (81)
White ethnicity, *n* (%)	40 (65)
Ethnicity, *n* (%)	Black—African 13 (21) Black—British 1 (2) Black—Caribbean 3 (5) Black—other 1 (2) Mixed—White and Black 3 (5) White—British 25 (40) White—Irish 9 (15) White—other 6 (10) Other 1 (2)
Identified sexuality, *n* (%)	Heterosexual 35 (56) Homosexual 20 (32) Unknown 7 (11)
Born in the UK or Ireland, *n* (%)	37 (60)
Continent of birth, *n* (%)	Europe 41 (66) Africa 13 (21) South America 2 (3) Asia 1 (2) Unknown 5 (8)
Time living in long-term care: years (median; range)	3 (0.5–19)
HIV history	
Time since HIV diagnosis: years (median; range)	21 (1–40)
HIV RNA < 50 copies/mL, *n* (%)	57 (92)
Nadir CD4 (cells/µL) (median; IQR)	154 (276)
Current CD4 (cells/µL) (median; IQR)	519 (359)
CD4:CD8 ratio (median; IQR)	0.55 (0.51)
Previous AIDS defining condition, *n* (%)	24 (39)
Co-morbidities	
Charlson Comorbidity Index: n (median; range)	5 (0–11)
Clinical Frailty Scale, *n* (%)	4—vulnerable 4 (6) 5—mildly frail 18 (29) 6—moderately frail 14 (23) 7—severely frail 18 (29) 8—very severely frail 6 (10) 9—terminally ill 2 (3)
Dementia diagnosis, *n* (%)	22 (35)
Medication	
Duration of ART: years (median; range)	17.5 (1–33)
Current ART-based regimen *n* (%)	NRTI 60 (97) NNRTI 8 (13) PI 16 (26) INI 46 (74)
Polypharmacy diagnosis, *n* (%)	56 (90)
Number of non-ART medications: *n* (median; range)	9 (0–18)
Anticholinergic Burden Scale score: *n* (median; range)	3 (0–14)
Anticholinergic Burden Scale score: *n* (%)	0—None 12 (19) 1—Mild 9 (15) 2—Moderate 9 (15) 3 +—Severe 32 (52)
Recent HIV care	
Total number of HIV consultations in last 12 months: *n *(median; range)	4 (0–28)
Number of HIV consultations conducted face to face in last 12 months: *n* (median; range)	2 (0–16)
Number of HIV viral load tests in last 12 months: *n* (median; range)	2 (0–9)

*ART* antiretroviral therapy, *INI* integrase inhibitor, *IQR* interquartile range, *NNRTI* nonnucleoside reverse transcriptase inhibitors, *NRTI* nucleoside/nucleotide reverse transcriptase inhibitors, *PI* protease inhibitors

### Demographics

The median age was 61 years (range 33–92). Men comprised 81% of the sample. Most participants identified as White ethnicity, and the majority (60%) were born in the UK or Ireland. Heterosexuality was the most common sexual orientation (56%). Participants had been living in a care home for a median of 3 years (range 0.5–19).

### HIV history

The median number of years living with HIV was 21 (range 1–40). Most participants (92%) were virally suppressed, with only one person having a viral load > 200 copies/mL. The median current CD4 count was 519 cells/µL (interquartile range 359). The median CD4:CD8 ratio was 0.55 (interquartile range 0.51). The median nadir CD4 count was 154 cells/µL (interquartile range 276), and 39% of participants had a documented previous AIDS-defining condition.

### Co-morbidities

Multimorbidity was common. The median CCI score was 5 (range 0–11) with 53% of the cohort having a CCI score of 5 or more. Frailty was prevalent: 94% of participants had a CFS score of 5 or more, indicating at least mild frailty. A formal diagnosis of dementia was recorded in 35% of residents.

### Medication

Participants had been taking ART for a median of 17.5 years (range 1–33). Almost all regimens included a nucleoside reverse transcriptase inhibitor (97%) and/or an integrase inhibitor (74%). Polypharmacy was common: 90% of residents were prescribed five or more non-ART medications, with a median of 9 (range 0–18) non-ART drugs. Hyper-polypharmacy (ten or more non-ART medications) was also common, occurring in 48% of participants. The median ACB score was 3 (range 0–14), and 52% of people had an ACB score ≥ 3, indicating a high anticholinergic burden.

### Recent HIV care

The median number of contacts with HIV services in the preceding 12 months was 4 (range 0–28). When only in-person consultations (clinic or community-based) were considered, the median fell to 2 (range 0–16). The median number of HIV viral load tests performed in the previous 12 months was 2 (range 0–9). However, 6% had no viral load testing and 24% had only one test during this period. Amongst the 19 participants with less than two viral load tests, 4 (21%) were on protease inhibitor–containing regimens. Overall, 10% of participants had only one recorded contact with their HIV service in the prior year and 3% had no contact, despite being registered in care.

### Provision of care to people residing in care homes

None of the centres had local guidelines specifically addressing HIV care for care home residents, and models of care varied across sites. Four centres allocated a regular clinician (doctor or nurse specialist) to service users including care home residents, while others used a more flexible approach. Most services offered clinic consultations with either a doctor or nurse specialist. At the Ian Charleson Day Centre (ICDC) and Harrison Wing, consultations were exclusively physician led; the Number 22 Clinic was entirely nurse led, with all care home residents reviewed in the community. At the remaining centres, consultations took place either in clinic (in person or via telephone/video) or as in-person visits to the care home. At all sites, community visits were undertaken by nurse specialists, with ICDC additionally providing visits by a HIV geriatrician. Four sites reported that, since the COVID-19 pandemic, care home residents have continued to be managed virtually at higher rates than before the pandemic and compared with non-care home residents. Two sites participated in external, community-based multidisciplinary team (MDT) meetings, whilst four had internal meetings to discuss complex cases. The remainder had no formal mechanism to discuss complex cases including care home residents.

## Discussion

Compared with national data from England and Wales, the age and gender distribution of our cohort differs from the general care home population where most residents are older and female [10]. Only 40% of participants in the evaluation were aged ≥ 65 years, compared with 82.1% of care home residents in the general population and 81% were male [10]. The predominance of White ethnicity in our cohort reflects both local HIV epidemiology and the broader care home population [10]. Many participants have lived with HIV for over two decades, with low nadir CD4 counts and a high prevalence of previous AIDS-defining illnesses, consistent with a group whose HIV was diagnosed in earlier treatment eras. As successive generations of people living with HIV age, the characteristics of those entering long-term care may change highlighting the need for repeated evaluations.

Multimorbidity was common in this cohort, with a median CCI score of 5 [15, 22]. Prior work has described clusters of multimorbidity in people living with HIV such as metabolic, mood/respiratory and substance use-related patterns and several of these conditions are incorporated within the CCI [15, 22]. In our evaluation, the CCI provided a pragmatic way of summarising comorbidity burden using routinely collected data. However, we did not formally examine multimorbidity patterns, and our data does not allow us to determine whether residents’ comorbidities fall into the previously described clusters [22]. A previous study of care home residents (n = 2727) found that 7% had a CCI ≥ 5 [31]. In our cohort a larger proportion (53%) met this threshold, but direct comparisons should be interpreted with caution given our small sample size and differences in age distribution.

Frailty was highly prevalent, with almost all residents (94%) classified as frail (CFS ≥ 5). Reported frailty prevalence is around 11% amongst community-dwelling people living with HIV and 52% among care home residents in recent meta-analyses [5, 23]. Our findings indicate a high level of vulnerability to frailty amongst care home residents living with HIV. Formal comparison with previous studies is difficult due to small, varied sample sizes and differences in frailty measurement tools used in those studies [5, 23]. The CFS is widely used in the UK and Ireland, is relatively simple to apply using routine clinical information and has demonstrated associations with mortality and functional/cognitive decline [16, 17, 24]. Validation work suggests that the CFS has acceptable construct validity in people living with HIV [25]. Our data supports the feasibility of using CFS in this context, but we did not compare CFS with other frailty measures such as the Fried Frailty Phenotype, which is the most common tool used in people living with HIV, so we cannot draw conclusions about the optimal frailty tool for this population [5, 26, 27].

A formal diagnosis of dementia was recorded in 35% of participants which appears lower than estimates of dementia prevalence in care home populations overall [28]. Possible explanations include differences in age given our younger cohort or under-recognition or under-recording of dementia. Our evaluation was not designed to explore indications for long-term care placement so reasons such as physical disability or neuropsychiatric conditions not coded as dementia may have been more common reasons.

Polypharmacy was also common with 90% of participants taking five or more non ART medications and 48% meeting the criteria for hyper-polypharmacy (ten or more medications) [29]. Polypharmacy in the general HIV population is approximately 44% in the Americas and 29% in Europe [30]. In a population analysis of 4,023 nursing home residents (57 care homes in 8 countries) participating in the Services and Health for Elderly in Long TERm care (SHELTER) project, a rate of 49.7% for polypharmacy and 24.3% for hyper-polypharmacy was seen [31]. These differences may reflect the intersection of ageing, HIV and multimorbidity, but formal comparative studies are required.

The ACB range was 0–14, with 52% of participants having a severe ACB score (≥ 3). In a sample of people living with HIV (*n* = 790) who attended the ‘over 50’s clinic’ at Chelsea and Westminster Hospital in London, 14.6% were on at least one anticholinergic drug compared to 82% in our sample [32]. This proportion is also greater than that reported in the general care home population [33]. Anticholinergic medications are associated with cognitive impairment, falls, frailty and mortality, and may have a particularly pronounced neuropsychological impact in people living with HIV [34–37]. Interventions to reduce anticholinergic burden in older adults have shown mixed results, highlighting the challenges of deprescribing in the context of multimorbidity [38]. Given the relatively young age of many participants in this cohort and the high prevalence of polypharmacy and anticholinergic burden, there is understandable concern about future cognitive trajectories as individuals age further. Whilst our cross-sectional data cannot address causality or long-term outcomes, they underscore the importance of regular medication reviews with specific focus on ACB ideally with input from clinical pharmacists experienced in deprescribing in the context of HIV [39].

There was substantial heterogeneity in how HIV services organised care for people living with HIV residing in long-term care. Some sites had regular named clinicians, others more flexible allocations; some conducted most reviews in the outpatient clinic, others predominantly in the community. No site had local guidelines specifically addressing care home residents, and formal cross-sector MDT structures varied. In our sample, the median number of contacts with HIV services over the preceding 12 months did reduce from four (range 0–28) to two (range 0–16) when only in-person reviews were considered, but this concordant with national guidance [40, 41]. However, 10% of participants had had only one contact with their HIV service over the preceding 12 months, whilst 3% had had no contact at all [40, 41]. For virally suppressed people living with HIV-1, the British HIV Association (BHIVA) recommends viral load testing to be performed at least 6-monthly, but can be extended to annually if a person is on an ART regimen that includes a protease inhibitor (PI) [41].In our sample, the median number of HIV viral load tests was 2 (range 0–9). However, 6% had no viral load testing, whilst 24% had only one in the last 12 months. Of the 19 people with less than two viral load tests, only 21% were on PI therapy that would permit reduced monitoring, Our data cannot distinguish between appropriate individualised decisions (e.g. reduced monitoring in those with very stable disease and significant multimorbidity) and gaps in recommended care, but it highlights potential vulnerability to reduced monitoring for care home residents. Previous work in other settings has suggested that extending the interval between in-person reviews for very stable people living with HIV may deliver time, financial and environmental efficiencies without compromising outcomes [42]. Our evaluation was not designed to assess cost or sustainability, and we did not model the impact of alternative monitoring schedules for care home residents. Nevertheless, future research could explore how visit frequency, mode of consultation (e.g. community visits vs clinic vs telemedicine) and MDT involvement influence outcomes for this group.

There was variability on how complex cases were managed between centres with some having no platform for discussion. Others had internal meetings which could leading to fragmentation of care, as they do not involve primary care physicians, care home staff and other relevant healthcare professionals. Some centres did engage with external MDT meetings. These meetings are an opportunity to discuss a person’s case with all involved healthcare professionals to ensure holistic joined up care whilst also providing an opportunity for education around what role, if any, HIV or ART may be contributing. Evidence shows positive attendees’ experiences of these meetings but there is limited evidence on long-term outcomes [43, 44]. HIV services may benefit from having internal processes in place to discuss complex cases whilst also participating in existing external MDT meetings. Likewise, there may be value in primary care or geriatric medicine teams involving HIV clinicians in discussions when the care of a person living with HIV is being considered.

The current literature suggests that knowledge of HIV amongst care home staff and geriatricians may be limited [45, 46]. Meanwhile, whilst primary care physicians generally feel confident managing multimorbidity in people living with HIV, they are perceived by people living with HIV as less knowledgeable about HIV itself [47–49]. People living with HIV also anticipate high rates of stigma and/or discrimination when accessing primary care services [42, 48–50]. Our findings of variable service models and absence of specific local guidelines support the need for further work on how best to integrate HIV, geriatric and primary care for residents in long-term care, while maintaining person-centred, goal-consistent care rather than rigid standardisation.

This evaluation has several strengths. It is, to our knowledge, the first multi-centre description of care home residents living with HIV across the UK and Ireland, and one of the earliest such cohorts reported in Europe. We used routinely collected data and established measures for multimorbidity, frailty, polypharmacy and anticholinergic burden, enhancing reproducibility and comparability [15–19]. However, important limitations must be acknowledged. Firstly, the sample size is small and heterogeneous, combining both younger residential care residents and older nursing home residents. Meanwhile, as the evaluation was descriptive and cross-sectional and we did not compare outcomes between models of care we cannot infer causality. Findings are not generalisable beyond similar high-income settings and should be interpreted cautiously. Secondly, data was derived from routine electronic records and local databases, so findings are contingent on the quality and completeness of documentation. Indication for entering long-term care (e.g. physical disability, acquired brain injury, mental health problems, substance use, previous homelessness) were not systematically captured and could not be reliably analysed. This is a key limitation, particularly given the relatively young median age compared with general care home populations and is an important area for future research [10]. Thirdly, the CFS was applied retrospectively based on clinical documentation rather than prospective assessment, and other frailty tools were not used for comparison [16, 17, 26, 27]. Similarly, whilst CCI, polypharmacy thresholds and ACB scores are well-established, we did not explore participants’ priorities, quality of life or functional trajectories, which are central to person-centred care. Finally, whilst we compared some descriptive findings with existing literature (e.g. SHELTER and other cohorts), differences in age structure, measurement tools and health systems limit the extent to which such comparisons can support generalisable conclusions [31, 33].

## Conclusions

In this service evaluation across seven centres in the UK and Ireland, care home residents living with HIV represented a small proportion of all people accessing HIV services but had high levels of multimorbidity, frailty, polypharmacy and anticholinergic burden. Models of HIV care for this group were heterogeneous and developed in the absence of specific local guidelines. As the number of people ageing with HIV increases, it is likely that more individuals will require long-term care. Descriptive studies such as theis provided a foundation to highlight potential areas of vulnerability and inform the design of future studies. Larger, adequately powered studies are needed to examine care models, monitoring strategies and outcomes for care home residents living with HIV and to support the development of evidence-informed guidelines, through collaboration between HIV, geriatric, primary and social care services.

## Supplementary Information

Below is the link to the electronic supplementary material.

Supplementary file1 (DOCX 20 KB)

## Data Availability

The data that support the findings of this study are available from the corresponding author upon reasonable request.

## References

[1] Wing EJ (2016) HIV and aging. Int J Infect Dis 53:61–68 27756678 10.1016/j.ijid.2016.10.004

[2] UK Health Security Agency. HIV testing, PrEP, new HIV diagnoses, and care outcomes for people accessing HIV services: 2022 report. 2022.

[3] Tavoschi L, Gomes Dias J, Pharris A (2017) New HIV diagnoses among adults aged 50 years or older in 31 European countries, 2004-15: an analysis of surveillance data. Lancet HIV 4(11):e514–e521 28967582 10.1016/S2352-3018(17)30155-8

[4] HIV Commission. How England will end new case of HIV – final report and recommendations. HIV Commission; 2020 [cited 2020 June 26th]; Available from: https://www.hivcommission.org.uk/wp-content/uploads/2020/12/HIV-Commission-Full-Report_online_final_pages.pdf.

[5] Jones HT, Levett T, Barber TJ (2022) Frailty in people living with HIV: an update. Curr Opin Infect Dis 35(1):21–30 34799510 10.1097/QCO.0000000000000798

[6] Nyblade L, Stangl A, Weiss E, Ashburn K (2009) Combating HIV stigma in health care settings: what works? J Int AIDS Soc 12(1):15 19660113 10.1186/1758-2652-12-15 PMC2731724

[7] Kall M, Marcellin F, Harding R, Lazarus JV, Carrieri P (2020) Patient-reported outcomes to enhance person-centred HIV care. Lancet HIV 7(1):e59–e68 31776101 10.1016/S2352-3018(19)30345-5

[8] OECD, Eurostat and World Health Organization. A System of Health Accounts 2011: Revised edition. OECD EU, World Health Organization, editor. Paris: OECD Publishing; 2017.

[9] Schols JMGA, Gordon A. Long-term care: residential and nursing home care; from the past to the future. In: Michel J-P, Beattie BL, Martin FC, Walston J, editors. Oxford Textbook of Geriatric Medicine: Oxford University Press; 2017.

[10] Office for National Statistics. Older people living in care homes in 2021 and changes since 2011. ONS; 2023 [cited 2024 July 27th]; Available from: https://www.ons.gov.uk/peoplepopulationandcommunity/birthsdeathsandmarriages/ageing/articles/olderpeoplelivingincarehomesin2021andchangessince2011/2023-10-09#cite-this-article.

[11] European AIDS Clinical Society. EACS Guidelines 2023. EACS; 2023 [cited 2024 October 1st]; Available from: https://www.eacsociety.org/media/guidelines-12.0.pdf.

[12] Jones HT, Samji A, Cope N, Williams J, Swaden L, Katiyar A et al (2022) What problems associated with ageing are seen in a specialist service for older people living with HIV? HIV Med 23(3):259–267 34693618 10.1111/hiv.13193

[13] Levett T, Roberts J, Adler Z, Wright J, Vera JH, Alford K (2020) Evaluation of a combined hiv and geriatrics clinic for older people living with hiv: The silver clinic in brighton, uk. Geriatr (Switzerland) 5(4):1–12 10.3390/geriatrics5040081 PMC7709685 33086666

[14] UK Health Security Agency. HIV: surveillance, data and management. 2014 [updated 17th February 2025; cited 2025 17th June]; Available from: https://www.gov.uk/government/collections/hiv-surveillance-data-and-management.

[15] Charlson ME, Pompei P, Ales KL, MacKenzie CR (1987) A new method of classifying prognostic comorbidity in longitudinal studies: development and validation. J Chronic Dis 40(5):373–383 3558716 10.1016/0021-9681(87)90171-8

[16] Rockwood K, Theou O (2020) Using the clinical frailty scale in allocating scarce health care resources. Can Geriatr J 23(3):210–215 32904824 10.5770/cgj.23.463 PMC7458601

[17] Rockwood K, Song X, MacKnight C, Bergman H, Hogan DB, McDowell I et al (2005) A global clinical measure of fitness and frailty in elderly people. CMAJ 173(5):489–495 16129869 10.1503/cmaj.050051 PMC1188185

[18] Masnoon N, Shakib S, Kalisch-Ellett L, Caughey GE (2017) What is polypharmacy? A systematic review of definitions. BMC Geriatr 17(1):230 29017448 10.1186/s12877-017-0621-2 PMC5635569

[19] Boustani M, Campbell N, Munger S, Maidment I, Fox C (2008) Impact of anticholinergics on the aging brain: a review and practical application. Aging Health 4(3):311–320

[20] Kiesel EK, Hopf YM, Drey M (2018) An anticholinergic burden score for German prescribers: score development. BMC Geriatr 18(1):239 30305048 10.1186/s12877-018-0929-6 PMC6180424

[21] King R, Rabino S. ACB Calculator. 2024. p. https://www.acbcalc.com/.

[22] De Francesco D, Sabin CA, Reiss P (2020) Multimorbidity patterns in people with HIV. Curr Opin HIV AIDS 15(2):110–117 31644480 10.1097/COH.0000000000000595

[23] Kojima G (2015) Prevalence of frailty in nursing homes: a systematic review and meta-analysis. J Am Med Dir Assoc 16(11):940–945 26255709 10.1016/j.jamda.2015.06.025

[24] Deng Y, Sato N (2024) Global frailty screening tools: review and application of frailty screening tools from 2001 to 2023. Intractable Rare Dis Res 13(1):1–11 38404737 10.5582/irdr.2023.01113 PMC10883846

[25] McMillan JM, Gill MJ, Power C, Fujiwara E, Hogan DB, Rubin LH (2021) Construct and criterion-related validity of the clinical frailty scale in persons with HIV. J Acquir Immune Defic Syndr 88(1):110–116 34050103 10.1097/QAI.0000000000002736

[26] Fried LP, Tangen CM, Walston J, Newman AB, Hirsch C, Gottdiener J et al (2001) Frailty in older adults: evidence for a phenotype. J Gerontol A Biol Sci Med Sci 56(3):M146–M156 11253156 10.1093/gerona/56.3.m146

[27] Fried LP, Ferrucci L, Darer J, Williamson JD, Anderson G (2004) Untangling the concepts of disability, frailty, and comorbidity: implications for improved targeting and care. J Gerontol A Biol Sci Med Sci 59(3):255–263 15031310 10.1093/gerona/59.3.m255

[28] Seitz D, Purandare N, Conn D (2010) Prevalence of psychiatric disorders among older adults in long-term care homes: a systematic review. Int Psychogeriatr 22(7):1025–1039 20522279 10.1017/S1041610210000608

[29] Jokanovic N, Tan ECK, Dooley MJ, Kirkpatrick CM, Bell JS (2015) Prevalence and factors associated with polypharmacy in long-term care facilities: a systematic review. J Am Med Direct Assoc 16(6):535 10.1016/j.jamda.2015.03.003 25869992

[30] Danjuma MI, Adegboye OA, Aboughalia A, Soliman N, Almishal R, Abdul H et al (2022) Prevalence and global trends of polypharmacy among people living with HIV: a systematic review and meta-analysis. Ther Adv Drug Saf 13:20420986221080796 36052397 10.1177/20420986221080795 PMC9425890

[31] Onder G, Liperoti R, Fialova D, Topinkova E, Tosato M, Danese P et al (2012) Polypharmacy in nursing home in Europe: results from the SHELTER study. J Gerontol A Biol Sci Med Sci 67A(6):698–704 10.1093/gerona/glr233 22219520

[32] Mazzitelli M, Milinkovic A, Pereira B, Palmer J, Tong T, Asboe D et al (2019) Polypharmacy and evaluation of anticholinergic risk in a cohort of elderly people living with HIV. AIDS. 10.1097/QAD.0000000000002403 31764110 10.1097/QAD.0000000000002403

[33] Oudewortel L, van der Roest HG, Onder G, Wijnen VJM, Liperoti R, Denkinger M et al (2021) The association of anticholinergic drugs and delirium in nursing home patients with dementia: results from the SHELTER study. J Am Med Direct Assoc 22(10):2087–2092 10.1016/j.jamda.2021.05.039 34197793

[34] Cooley SA, Paul RH, Strain JF, Boerwinkle A, Kilgore C, Ances BM (2021) Effects of anticholinergic medication use on brain integrity in persons living with HIV and persons without HIV. AIDS. 10.1097/QAD.0000000000002768 33252494 10.1097/QAD.0000000000002768 PMC7855412

[35] Michael HU, Brouillette M-J, Tamblyn R, Fellows LK, Mayo NE (2024) Cognitive impact of anticholinergic and sedative burden in people with HIV. AIDS. 10.1097/QAD.0000000000003966 38905493 10.1097/QAD.0000000000003966

[36] Ali S, Peterson GM, Bereznicki LR, Salahudeen MS (2020) Association between anticholinergic drug burden and mortality in older people: a systematic review. Europ J Clin Pharmacol 76(3):319–335 10.1007/s00228-019-02795-x 31832732

[37] Doctor J, Winston A, Vera JH, Post FA, Boffito M, Mallon PWG et al (2023) Anticholinergic medications associated with falls and frailty in people with HIV. HIV Med 24(12):1198–1209 37644705 10.1111/hiv.13532

[38] Braithwaite E, Todd OM, Atkin A, Hulatt R, Tadrous R, Alldred DP et al (2023) Interventions for reducing anticholinergic medication burden in older adults—a systematic review and meta-analysis. Age Ageing. 10.1093/ageing/afad176 37740900 10.1093/ageing/afad176 PMC10517713

[39] Hill LA, Ballard C, Cachay ER (2019) The role of the clinical pharmacist in the management of people living with HIV in the modern antiretroviral era. AIDS Rev 21:4 31834321 10.24875/AIDSRev.19000089

[40] Budak JZ, Scott JD, Dhanireddy S, Wood BR (2021) The impact of COVID-19 on HIV care provided via telemedicine—past, present, and future. Curr HIV/AIDS Rep 18(2):98–104 33616811 10.1007/s11904-021-00543-4 PMC7898490

[41] British HIV Association. BHIVA guidelines for the routine investigation and monitoring of adult HIV-1-positive individuals (2019 interim update). BHIVA; 2019 [cited 2024 October 14th]; Available from: https://www.bhiva.org/file/DqZbRxfzlYtLg/Monitoring-Guidelines.pdf.

[42] Riddell L (2024) What if we see you in clinic once a year? Triple benefits of changing the HIV care pathway to align with other chronic diseases. HIV Med 25(S1):9–10

[43] Tan LF, Teng J, Chew ZJ, Choong A, Hong L, Aroos R et al (2023) Geriatric services hub: a collaborative frailty management model between the hospital and community providers. J Frailty Aging 12(4):316–321 38008983 10.14283/jfa.2023.23 PMC10111077

[44] Steel A, Hopwood H, Goodwin E, Sampson EL (2022) Multidisciplinary residential home intervention to improve outcomes for frail residents. BMC Health Serv Res 22(1):58 35022056 10.1186/s12913-021-07407-y PMC8756619

[45] Garley J, Bignell C, das Nair R (2014) A qualitative survey of attitudes towards HIV amongproviders of community care. HIV Med 15(S3):45

[46] Jones HT, Barber TJ (2022) How do geriatricians feel about managing older people living with HIV? A scoping review. Eur Geriatr Med 13(4):987–997 35397097 10.1007/s41999-022-00642-4 PMC9378329

[47] Defty H, Kennedy M, Smith H, Perry N, Fisher M (2010) GPs’ perceived barriers to their involvement in caring for patients with HIV: a questionnaire-based study. Br J Gen Pract 60(574):348–351 20423586 10.3399/bjgp10X501840 PMC2858532

[48] Petchey R, Farnsworth B, Williams J (2000) The last resort would be to go to the GP’. Understanding the perceptions and use of general practitioner services among people with HIV/AIDS. Soc Sci Med (1982) 50(2):233–245 10.1016/s0277-9536(99)00278-6 10619692

[49] Hawkins L, Cassell J (2015) What is the role of general practice and the potential barriers in providing shared care for people living with HIV: a systematic review. Sex Trans Infect 2015:91

[50] Brown MT, Bussell JK, editors. Medication adherence: WHO cares? Mayo clinic proceedings; 2011: Elsevier. 10.4065/mcp.2010.0575 PMC3068890 21389250

