# Supplementary material for: How are HIV services in the UK and Ireland managing care home residents living with HIV?
Source: Eur Geriatr Med. 2025 Dec 19;17(2):1029–37. doi: 10.1007/s41999-025-01389-4 (PMC13109281; doi:10.1007/s41999-025-01389-4)
Supplement: Supplementary file 1 — Supplementary file1 (DOCX 20 KB) [file 41999_2025_1389_MOESM1_ESM.docx]

**Appendix 1 – Data Collection Tool**

| Local ID | This will be 3 letters then a number based on each site |  |  |
| --- | --- | --- | --- |
| Age (years) | Age as of September 2024 in years |  |  |
| Gender (categorical) | Gender; any trans participants should be coded as their identified gender please but please make a note at the bottom of your sheet so we can determine how many trans/non-binary people there were |  |  |
| Ethnicity (categorical) | As coded locally as per HARS, if unknown please document as "Not recorded" |  |  |
| Country of birth | As coded locally as per HARS, if unknown please document as "Not recorded" |  |  |
| Sexuality (categorical) | As coded locally as per HARS, if unknown please document as "Not recorded" |  |  |
| Time since HIV diagnosis (years) | Number of years since diagnosis |  |  |
| Current HIV viral load (copies/mL) | Most recent HIVVL, if undetectable please use the lower limit from your centre e.g. at Royal Free we would say <40, please document at the bottom of your sheet what cut off you use, as far as write up we will consider <200 | | |
| Current CD4 (cell/mm3) | Most recent CD4 count |  |  |
| Nadir CD4 (cell/mm3) | Nadir CD4 if available, if if unknown please document as "Not recorded" |  |  |
| CD4:CD8 | Most recent ratio |  |  |
| Previous AIDS defining condition (yes/no) | As per HARS - Yes or No; if unknown please put No |  |  |
| Durantion on ART (Years) | Number of years since diagnosis |  |  |
| Current ART (free text) | Most recent ART prescribed |  |  |
| Number of non-ART medications | How many non ART medications recorded in record |  |  |
| ACB Score | Using the below website, enter all medication in to the calculator to determine a score, if a drug cannot be found that means it scores 0, ART will not be included |  |  |
| Charlson Comorbidity Index (0-37) | Using data from local records use the CCI to calculate a score of between 0 and 37, please note AIDS should only be ticked if current CD4 count of less than 200, not historically |  |  |
| Clinical Frailty Scale (0-9) | Use the link below to determine the frailty status |  |  |
| Dementia Diagnosis (yes/no) | Using data from local records record if there is a formal diagnosis of dementia, yes or no, if unknown please put no |  |  |
| Time in Care Home (years) | Using data from local records review all changes to address to determine when moved into a care home and calculated the number of years living in long-term care |  |  |
| Total number of consultations in last 12 months | The number of total contacts between the person and your HIV service including both telephone and face to face |  |  |
| Number of face-to-face consultations in last 12 months | Only face to face contacts in the last 12 months, can be in clinic or the community, by any care home staff |  |  |
| Number of HIV VL blood tests in last 12 months | The number of HIVVL taken on your system in the last 12 months from the date of your data collection |  |  |

ART – Antiretroviral Therapy; HARS - HIV and AIDS Reporting System, HIVVL – HIV viral load

**Websites Used**

Acetylcholine burden calculator - https://www.acbcalc.com/

Charlson Comorbidity Index - https://www.mdcalc.com/calc/3917/charlson-comorbidity-index-cci

Clinical Frailty Scale - https://www.acutefrailtynetwork.org.uk/uploads/files/1/CFS%20Quick%20Reference%20Guide%20FINAL.pdf

**Service Delivery Survey Questions**

| 1. Does your service have a standard operating procedure (SOP) on managing people living in care homes (Yes/No) | Yes or No |
| --- | --- |
| 2. Does one clinician oversee all care home residents or is it divided between staff | I.e. if someone is in a care home do they transfer to a single person or do you share them amongst the team |
| 3. If divided do care home residents have a named responsible clinician in the service or is each review with an available clinician | Do they see the same person each time or does it vary |
| 4. Are they a doctor (consultant, associate specialist, registrar) or are they a clinical nurse specialist, is it mixed? | Background of the people they have seen over last few visits |
| 5. Are the seen in clinic, in the care home or mixed | Where were the last few face to face reviews done home or community |
| 6. Since the Covid-19 pandemic do you feel have you been conducting more virtual reviews of these patients as opposed to face to face | Subjective view, are you doing more virtual consultations |
| 7. Do clinic staff attend any care home or community multidisciplinary team meetings e.g. local frailty hub meetings | E.g. frailty hub, complex patient management group meetings, nursing home MDT etc |
| 8. Does the service have an internal MDT meeting where patients with complexity are discussed (not-including any ART/resistance MDTs) | I.e. a complex MDT, frailty MDT that exists on top of the standard resistance MDT |
| 9. Has the clinic done anything else aimed to improve care for these patients | Any local strategies not covered above focused on care home residents |
| 10. Anything else relevant you feel you want to add |  |
